# The Influence of Enzymatic Hydrolysis of Whey Proteins on the Properties of Gelatin-Whey Composite Hydrogels

**DOI:** 10.3390/ma14133507

**Published:** 2021-06-23

**Authors:** Violeta Popescu, Andreia Molea, Marioara Moldovan, Pompilia Mioara Lopes, Amalia Mazilu Moldovan, George Liviu Popescu

**Affiliations:** 1 Physics and Chemistry Department, Technical University of Cluj-Napoca, 28 Memorandumului Str., 400114 Cluj-Napoca, Romania; violeta.popescu@chem.utcluj.ro (V.P.); Mioara.Lopes@im.utcluj.ro (P.M.L.); amalia.mazilu@gmail.com (A.M.M.); 2 Automotive Engineering and Transports Department, Technical University of Cluj-Napoca, 28 Memorandumului Str., 400114 Cluj-Napoca, Romania; Andreia.MOLEA@auto.utcluj.ro; 3 Institute of Chemistry Raluca Ripan, Babes-Bolyai University, 30 Fantanele Str., 400294 Cluj-Napoca, Romania; marioara.moldovan@ubbcluj.ro

**Keywords:** whey proteins isolate, whey proteins hydrolysates, hydrogels, swelling degree, swelling kinetics

## Abstract

Amino-acids, peptides, and protein hydrolysates, together with their coordinating compounds, have various applications as fertilizers, nutritional supplements, additives, fillers, or active principles to produce hydrogels with therapeutic properties. Hydrogel-based patches can be adapted for drug, protein, or peptide delivery, and tissue healing and regeneration. These materials have the advantage of copying the contour of the wound surface, ensuring oxygenation, hydration, and at the same time protecting the surface from bacterial invasion. The aim of this paper is to describe the production of a new type of hydrogel based on whey protein isolates (WPI), whey protein hydrolysates (WPH), and gelatin. The hydrogels were obtained by utilizing a microwave-assisted method using gelatin, glycerol, WPI or WPH, copper sulfate, and water. WPH was obtained by enzymatic hydrolysis of whey protein isolates in the presence of bromelain. The hydrogel films obtained have been characterized by FT-IR and UV-VIS spectroscopy. The swelling degree and swelling kinetics have also been determined.

## 1. Introduction

Amino-acids, peptides, and protein hydrolysates together with their coordinating compounds have various applications as fertilizers, nutritional supplements, additives, fillers, or active principles to produce hydrogels with therapeutic properties [1,2].

Whey is the soluble fraction of milk, rich in proteins, minerals, and lactose that are separated from casein during the manufacture of cheese or casein.

Whey has been considered for a long time as a by-product of cheese and curd manufacturing. In the last years, whey proteins and their hydrolyzed products have proved to have a large scale of bioactive properties.

Whey can be a very useful and valuable solution among protein raw materials for amino-acid/peptide production, due to its low cost and availability, high nutritional value, low bitterness and low antigenicity; whey being the major by-product of cheese manufacturing, representing up to 20% of total milk proteins [3,4].

Enzymatic hydrolysis of whey can be used for improving the functional, nutritional, and immunological properties of proteins, reducing the allergenicity and antigenicity of the proteins.

Bioactive components of the hydrolyzed products of whey cover a wide spectrum of compounds with anti-carcinogenic, antifungal, antihypertensive, antimicrobial [5], antioxidative [6,7], anti-proliferative, anti-thrombotic, antiulcerogenic [8], antiviral, and immunomodulatory [9] properties, while also having prebiotic activity [10,11,12].

Hydrogel-based patches can be adapted for drug, protein, or peptide delivery, and tissue healing and regeneration [13]. These materials have the advantage of copying the contour of the wound surface, ensuring oxygenation, hydration, and at the same time protecting the surface from bacterial invasion.

The biocompatibility of whey hydrogels and the exposure of the hydrophobic groups that can bind different types of drugs allows them to be used as drug delivery systems [14].

Gels or soft hydrogels obtained from proteins, peptides, or other natural compounds can also be used for tooth whitening and remineralization [15,16].

The aggregation of proteins and the properties of the gels, hydrogels, or microspheres based on whey can be influenced by other components such as k-carrageenan [17,18,19,20,21], alginate [22,23,24,25,26,27], gellan [28], chitosan [29], xanthan, pectine [27], carbonanotubes, and carbon nano-onions [30], most of which also have crosslinking effects.

Whey protein isolates and whey protein concentrates can be used as renewable raw materials for obtaining polymer sheets by free radical polymerization with poly(ethylene glycol) methyl ether methacrylate (PEGMA) [31] or tissue adhesive using glutaraldehyde [32].

Whey protein hydrogels can be obtained by ionotropically crosslinking with CaCl_2_ [29,33,34,35,36], MnCl_2_ and ZnCl_2_ [35], NaCl [28,36], Fe^2+^ [37,38] and Mg^2+^ [39] or chemically crosslinking with citric acid [40,41], D-(+)-Gluconic acid D-lactone (GDL) [42], isomaltooligosaccharide [43], and tanninic acid [14].

In order to improve the mechanical properties of whey protein hydrogels, B. A. Aderibigbe and S. Ndwabu obtained whey protein isolate-graft-carbopol-polyacrylamide-based hydrogel composites [44].

Although there are numerous studies on obtaining hydrogels based on gelatin [45,46,47,48] with application related to wound dressing and hydrogels based on whey proteins with other applications [49,50], the studies related to the hydrogels based on gelatin and whey proteins are quite rare, but the obtaining and characterization of diverse, composites or films has been studied [51,52,53,54,55].

Strong interactions between whey and gelatin determined important changes in the mechanical, thermal, and gelling properties of whey protein–gelatin composite films or gels [54,55,56,57,58]. 

The addition of small amounts of gelatin to WPI improves the strength and stability of gels, indicating synergistic interaction between whey and gelatin, in the presence or the absence of a crosslinking agent such as transglutaminase, influencing the rheological and thermal properties of materials [54,59,60] and water holding capacity [56]. The introduction of gelatin in composite laminated multilayered films containing WPI and sodium alginate increased the elasticity of the films [57]. The interaction between whey and gelatin depends on the pH and polymer ratio [58], depending also on the treatments applied to whey, for example, the polymerization of whey increased the interactions between the two proteins [59].

On the other hand, the addition of whey proteins conducted to the stabilization of gelatin and carboxymethylcellulose water-in-water emulsion [60] or aerated gelatin gels [61].

Whey protein isolates and porcine gelatin have been also successfully tested for incorporation of quinoa oil using Tween 20 as surfactant, making the oil more dispersible in water, and potentializing the antioxidant activity for application in a food matrix [62].

Although there are many studies related to the obtaining and characterization of gels or films, we have identified only one article that addresses the obtaining of a whey and gelatin hydrogel. This study involved an investigation on a multicomponent organic–inorganic WPI/gelatin/calcium phosphate hydrogel composites for bone tissue engineering [63].

We introduced copper ions into the hydrogel matrix because copper ions, its derivatives such as amino-acid complexes [64,65,66], copper nanoparticles [67,68], and nano-copper-zinc alloy (nCuZn) [69] have both functional and antibacterial properties.

The aim of this research is to produce a new type of hydrogel based on whey protein isolates or whey protein hydrolysates and gelatin crosslinked with copper ions. As far as we know, it is the first study that approaches hydrogels based on WPI or WPI hydrolysates and gelatin, crosslinked with copper ions.

## 2. Materials and Methods

### 2.1. Materials

Whey Protein Izolate (WPI) ISOLAC produced by Carbery Group and distributed by S.C. Way Better Nutrition from Cluj-Napoca has been used for the obtaining of whey protein hydrolysates using Bromelain from pineapple stem from BioChemica. Details regarding WPI main characteristics are presented in our paper [70]. A solution of 0.45 M/L NaOH (Sigma-Aldrich, Taufkirchen, Germany) has been used for maintaining the pH constant during the enzymatic hydrolysis applying pH-stat method [71]. Gelatin 270 bloom type A (Gelita, Cotia, São Paulo, Brazil), glycerol (Sigma-Aldrich, Taufkirchen, Germany), CuSO_4_ * 5H_2_O (Sigma-Aldrich, Taufkirchen, Germany) have been used for hydrogels preparation.

### 2.2. The Production of Whey Hydrolysates

Whey Protein Hydrolysates (WPH) obtained by enzymatic hydrolysis of solutions of 5% whey protein isolate (WPI), in the presence of 0.05% bromelain have been used to produce the hydrogels. For hydrolysis 200 mL solutions of WPI have been prepared using distilled water. For a better dispersion of the whey powder the solution was sonicated for 15 min and preheated to 60 °C and the pH was adjusted to 8. The hydrolysis took place for 360 min at constant pH with bromelain and the degree of hydrolysis determined based on the consumption of NaOH solutions with known concentration [71,72] was 3.92%.

### 2.3. Preparation of Hydrogels

Gelatin-based hydrogels were obtained by microwave assisted method using gelatin, glycerol, whey hydrolysates or WPI and CuSO_4_ * 5H_2_O. The gelatin was mixed with glycerol and water, then the sample was kept at room temperature for 10 min, followed by the addition of WPI or WPH solutions. The mixtures were subjected to irradiation in a household microwave system at 480 W for 25 s until homogenous and clear solutions were obtained.

To determine the influence of the cross-linking agent, hydrogels with different concentration of Cu^2+^ were synthesized from 0.5 g gelatin, 0.4 g glycerol, 4 mL hydrolysates or 5% WPI solutions, 2 mL water adding various quantities of 30% CuSO_4_ × 5H_2_O solutions. The samples were poured in Petri dishes and dried at room temperature for 2 days. For comparison, control hydrogel samples were prepared using 5% whey protein isolate solutions.

Table 1 presents the conditions for the obtaining of gelatin-whey hydrogels.

### 2.4. Optical Properties of the Hydrogels

Optical properties in visible and near infrared region of spectra (between 400 and 1000 nm) of the hydrogel films were determined using a double bean Perkin Elmer Lambda 25 UV/Vis Spectrophotometer (PerkinElmer, Waltham, MA, USA) before taking the samples from the Petri dishes, using for blank two clean empty Petri dishes.

### 2.5. Swelling Tests

For swelling tests, a method adapted from [73] has been applied. Tea bags were immersed in the swelling medium (distilled water) for 24 h. The empty bags were patted with paper towels to remove excess water and weighted (*W_1_*). Around 0.05 g of samples were weighted (*W_2_*), placed into the tea bag and sealed. The samples from tea bags were immersed in swelling media, weighted at predetermined times (*W_3_*) until the swelling equilibrium has been reached. The swelling degree [g water/g hydrogel] has been calculated with the equation:

(1) $$SW=\frac{W_{3}-W_{2}-W_{1}}{W_{2}}\left[g/g\right]$$

If SW is expressed as percent, the following equation must be used:

(2) $$SW\%=\frac{W_{3}-W_{2}-W_{1}}{W_{2}}\cdot 100\;[\%]$$

### 2.6. Thickness of the Films

The thickness of the films has been measured with a TROTEC BB25 coating thickness meter. For each sample, the thickness has been measured in 4 points and the average value has been calculated.

## 3. Results and Discussions

### 3.1. FTIR Analysis of WPI, WPH and Gelatin-Whey Hydrogels

The FT-IR spectra of WPI, WPH and hydrogels have been normalized for Amide I band (wavenumbers from 1800 to 1600 cm^−1^), to make a better comparison between them for evaluation of the changes into the protein’s structures.

**Figure 1:**
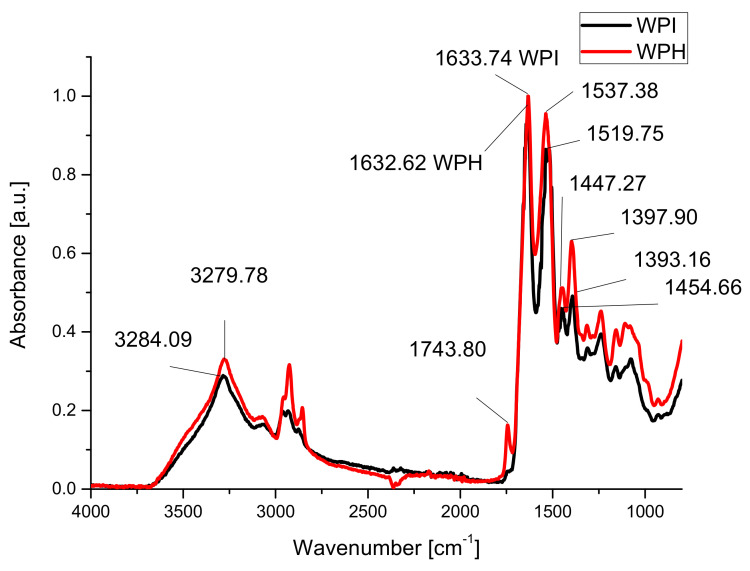
FTIR spectra of whey protein isolate (WPI) and whey protein hydrolysate (WPH).

FT-IR spectra of whey protein isolates and whey protein hydrolysates are presented in Figure 1.

FT-IR spectra showed an increase in the intensities of absorption band centered around 1536 cm^−1^ for the hydrolyzed sample compared to WPI due to the formation of primary amines following hydrolysis [74]. More than that, the band centered around 1400 cm^−1^ (related to terminal -COOH group) (amide III [75]) also increased compared to the band from 1632 cm^−1^ (amide I). A new absorption band appeared at around 1744 cm^−1^ due to the formation of new carboxylic groups following enzymatic hydrolysis. Compared to amide I absorption band one can see increased of the intensity and area of the vibration band (3640–3097 cm^−1^) due to the hydrogen bonds in the case of hydrolyzed sample.

In the case of hydrogels, FTIR analyses were carried out to observe changes into the protein’s structures following the formation of hydrogels in different conditions. The absorption bands amide I (centered at 1633 cm^−1^), amide II (around 1537 cm^−1^) and amide III (between 1400 and 1200 cm^−1^) [41,76] have been analyzed for studying changes into the secondary structure of proteins, while the absorption band from 1034 cm^−1^ due to the stretching vibration of hydroxyl groups of glycerol was studied in order to evaluate the interactions with the plasticizer [35,43].

Amide I absorption band shifts to smaller wavenumbers, 1632.62 cm^−1^ compared to 1633.74 cm^−1^ for sample WPH due to the disruption of C=O· · ·H–N hydrogen bonds in whey following hydrolysis, and can be associated with the decreasing of the α helix structures in the proteins [77,78].

**Figure 2:**
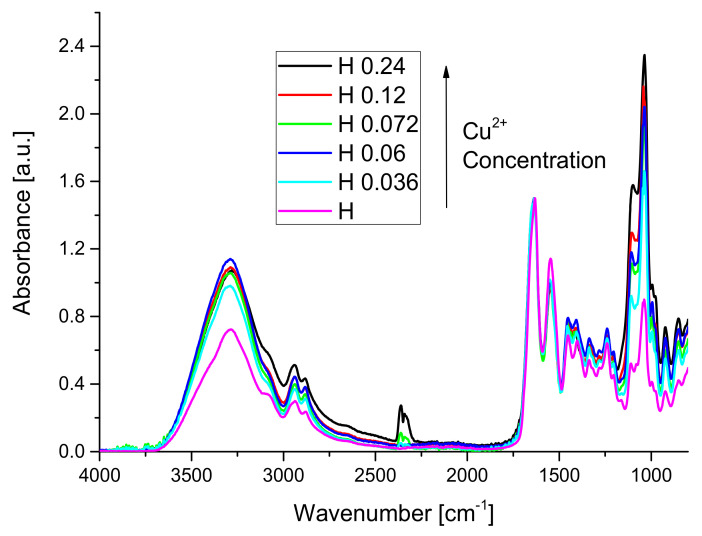
The influence of copper concentration on the FT-IR spectra of hydrogels.

The influence of copper concentration of the FT-IR spectra of hydrogels is presented in Figure 2.

Comparing the spectrum of WPH with the spectra of hydrogels, one can observe that the absorption band from 1744 cm^−1^ disappeared from the spectra of hydrogels because copper interacted with –COOH groups of whey forming ionic crosslinking bridges between the protein chains. No new absorption bands appeared in the spectra of hydrogels because no new covalent bonds were formed. Important changes in the amide A, amide II, amide III absorption bands have been observed. Amide A band, which can be attributed to NH stretching coupled with hydrogen bonds, suffered an important increase due to the presence of the copper sulfate in the sample. The peak situated around 1033 cm^−1^ is related to the interactions arising between the plasticizer (OH group of glycerol) and film structure [41,78].

**Figure 3:**
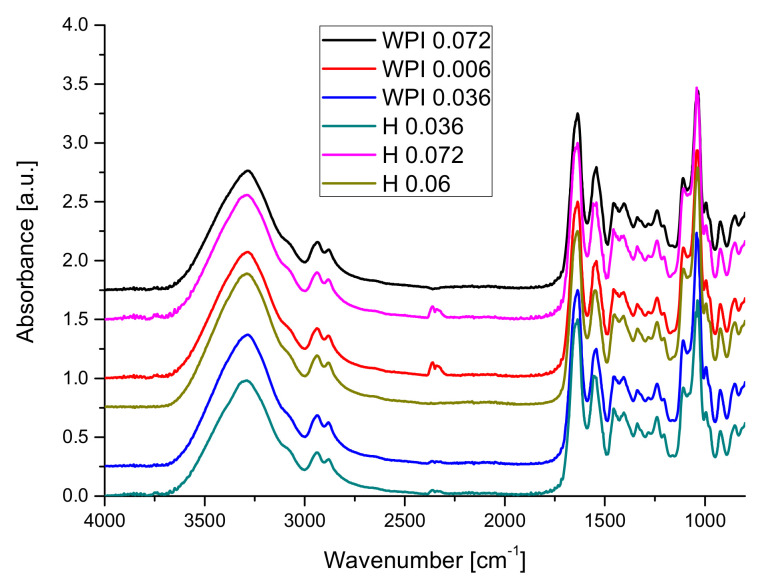
The influence of the WPH on of the FT-IR spectra of hydrogels.

The influence of using hydrolyzed whey protein on FT-IR spectra is presented in Figure 3.

Figure 3 illustrates that the use of hydrolyzed whey protein leads to an increase in intensity of the hydrogen bond vibrations (samples 0.06 and 0.072) leading also to small changes in amide I bands and important changes in amide II and amide III bands and the band from 1033 cm^−1^. Changes in the amide III vibrations band may be related to changes in the secondary structure of proteins chains following the addition of copper [79,80].

One can conclude that the hydrolysis process exposed more functional groups able to form hydrogen bonds in the hydrogels and to influence the interaction between the protein’s chains and plasticizer.

The increasing number of hydrogen bonds will lead to increasing of stability of hydrogels.

### 3.2. Optical Properties of the Hydrogels

Proteins from gelatin and whey and their hydrogels absorb small wavelengths in the UV region of the spectrum, presenting an absorption peak at 280 nm [81] and low absorption in visible and near infrared. Globular proteins from whey and gelatin absorb wavelengths from UV region of the spectrum in the range 190–220 nm due to peptide bonds and amino-acids and around 280 nm due to absorption of tyrosine and tryptophan [81].

**Figure 4:**
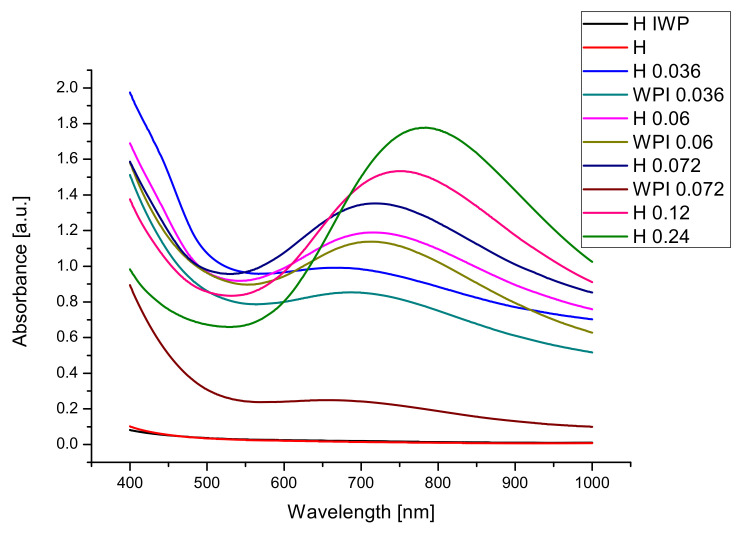
Optical properties of hydrogel films.

For our sample, the optical properties in the wavelengths of the visible range (Figure 4) were of main interest because of the intended application of the hydrogels as wound healing dresses or drug delivery transdermal patches.

The samples with the smallest absorption are the samples obtained from the gelatin and whey protein hydrolysates without Cu(II) crosslinking agent.

Using copper sulphate for obtaining hydrogels leads to the formation of samples with higher absorbance, in all cases. The high values of absorbance for films containing copper can be explained by the presence of the copper-based crosslinking agent. For facilitating the study of our hydrogels, we prepared thick samples, but for clinical application thinner films will be made to allow visual observation of the treated area. By increasing the concentration of Cu^2+^ films with smaller transmittance (higher absorbance) have been obtained. The use of hydrolyzed whey proteins led to an increase in the absorbance of the film, due to changes in the structure of the hydrogels.

### 3.3. Swelling Tests

**Figure 5:**
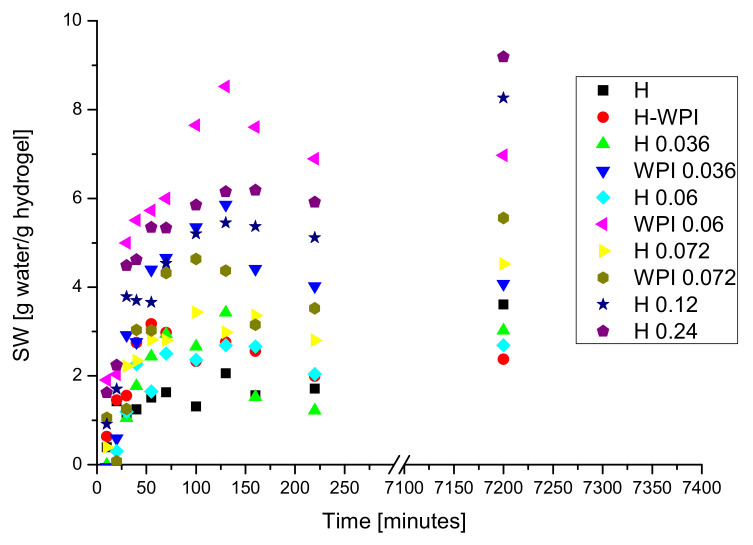
The variation of the swelling degree as a function of swelling time.

The results obtained for swelling degree are presented in Figure 5.

The swelling process took place with a high rate the first 50–100 min. The presence of copper ions as crosslinking agent and the hydrogen bonds formed between the proteins chains involving both functional groups from proteins and plasticizer (glycerol) stabilized the hydrogel that continue to swell, at a smaller rate when the materials were kept in water for 120 h. The turbidity of swelling environment increased following the swelling experiment suggesting that a part of hydrogel matrix begins to disintegrate, phenomenon worth investigating into the future. The decreasing of the swelling rate revealed changes in the swelling dynamics.

Swelling kinetics in water of hydrogels can be evaluated with the relation:

(3) $$\frac{W_{t}}{W_{e}}=k_{F}\cdot t^{n_{F}}$$

where: *W_t_* is the weight of hydrogel at time *t*, while *W_e_* is the weight of hydrogel at equilibrium. *K_F_* is diffusion constant of water into the hydrogel matrix, and n is an exponent of the diffusion process. If *n_F_* = 0.5, the diffusion process takes place based on a Fickian perfect model, when the rate of the relaxation of the polymer is higher than the rate of water diffusion. The value of *n* = 1 indicated a non-Fickian diffusion process when the diffusion rate of water is higher than the rate of relaxation of polymeric chains. If 0.5 < *n* < 1 relaxation rate of the network is comparable with the rate of water diffusion. The exponents of the swelling process for hydrogels are calculated from the slope of the chart $\log\left(\frac{W_{t}}{W_{e}}\right)=f\left(logt\right)$ for swellings smaller than 60% [42,82,83]. For our samples the correlation coefficients for the plots $\log\left(\frac{W_{t}}{W_{e}}\right)=f\left(logt\right)$, for all swelling time (plot not presented) have small values revealing that the whole process does not follow a Fickian model. However, in the first 30 to 55 min of swelling, depending on the sample, the swelling follows a Fickian model, and the values of constant *n_F_* are between 0.5 and 1 for some samples, suggesting that relaxation rate of the network is comparable with the rate of water diffusion, or higher than 1 for other samples suggesting non-Fickian kinetics. The values obtained for *k_F_, n_F_*, and R_F_^2^ from the plot $\log\left(\frac{W_{t}}{W_{e}}\right)=f\left(logt\right)$ are presented in Table 2. There are no clear correlations between the composition of the hydrogels and the values of constants obtained from the Fickian model.

Similar behavior has been reported for hydrogels based on whey protein concentrates loaded with caffeine [82]. They concluded that swelling curves deviate from the classical Fickian model because the swelling process is not a passive diffusion involving the penetration of solvent into the pores of the hydrogels network, but includes a simultaneous relaxation of the protein’s chains conducting to a significant increase in volume.

Another Fickian equation that described the diffusion of swelling medium molecules through hydrogel taking into consideration the swelling degree (*SW*), diffusion constant (*k*) of water into the hydrogel matrix, diffusion exponent (*n*) can be used for kinetic studies:

(4) $$\;\;SW=k\cdot t^{n}$$

If there is a linear relationship between *SW_t_* and *t*^1/2^, the process follows a Fickian model.

The fitting curves $SW_{t}=f\left(t^{\frac{1}{2}}\right)$ for the prepared hydrogels (not presented) presents no linearity, demonstrating once again that the process does not follow a Fickian model.

If global the process follows second order kinetics, the following equation describes the process [83]:

(5) $$\frac{dSW}{dt}=k\cdot {\left(SW_{e}-SW_{t}\right)}^{2}$$

where: $\frac{dSW_{t}}{dt}$ is the swelling rate, *k* is the rate constant (1/h), and *SW_e_* is the value of the swelling at equilibrium (maximum swelling degree).

By integration and rearrangement of the terms, Schott’s equation results [73]:

(6) $$\frac{t}{\mathrm{SW}}=\frac{1}{k\cdot {\mathrm{SW}}_{e}^{2}}+\frac{t}{{\mathrm{SW}}_{e}}$$

By plotting $\frac{t}{SW}$ against t a straight line with the slope of $\frac{1}{SW_{e}}$ and the intercept of $\frac{1}{k}\cdot SW_{e}^{2}$ will be obtained. The value of *SW_e_* will be calculated from the slope and the value of diffusion constant k from the intercept or the Schott’s equation:

(7) $$\;\mathrm{SW}=\frac{1}{\mathrm{slope}}$$

(8) $$\;k=\frac{1}{\mathrm{Intercept}\cdot {\mathrm{SW}}_{e}^{2}}$$

**Figure 6:**
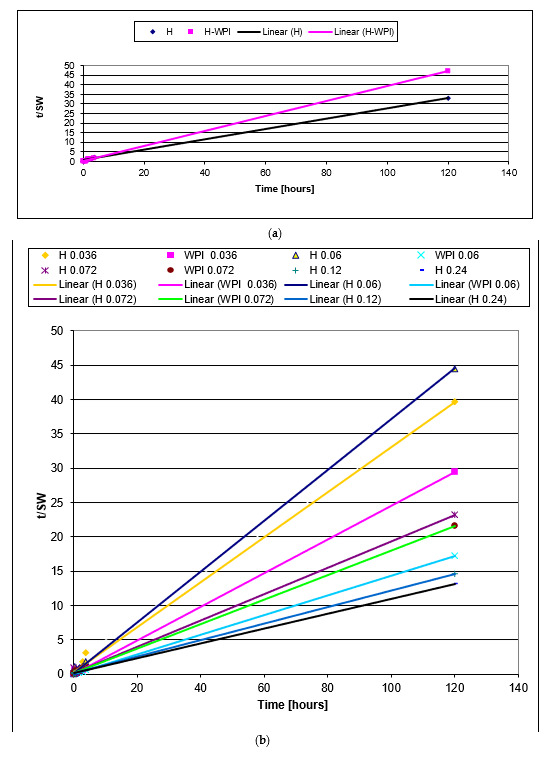
The plot of t/SW against t for samples H and WPI (**a**), and samples containing copper (**b**).

The plot of $\frac{t}{SW}$ against t for samples H and WPI are presented in Figure 6a, while for samples containing copper in Figure 6b. In all cases, a straight line was obtained with high values for correlation coefficients.

Sundaram Gunasekaran et al. showed that whey protein hydrogels are pH sensitive and the swelling kinetics depend on pH [44,82]. They concluded that raising the pH of the swelling medium increases the swelling rate.

The dependence of calculated swelling degree, experimental value of swelling degree, and rate constant as a function of copper concentration is presented in Table 2.

One can see that for samples that contain more than 0.87% CuSO_4_ * 5H_2_O, both calculated and experimental values for swelling degree increased with increasing copper concentration. Increasing the number of crosslinking bridges due to ionic bonds determined an increase in the stability of hydrogels. For almost all samples, the value of calculated maximum swelling degree is a little higher than the value of the experimental one, due to the dissolution of the sample into the swelling environment when the SW was high.

Rate constant k, decreased with increasing concentration of copper (II) ions due to the increasing of crosslinking process by Cu^2+^ bridges.

For all samples, the swelling degree decreased when WPH have been used instead of WPI demonstrating the importance of the new functional groups formed during enzymatic hydrolysis.

The value of the rate constant decreased with the increase in copper concentration. The highest rate constant value has been obtained in the case of the sample made from WPI without copper.

**Figure 7:**
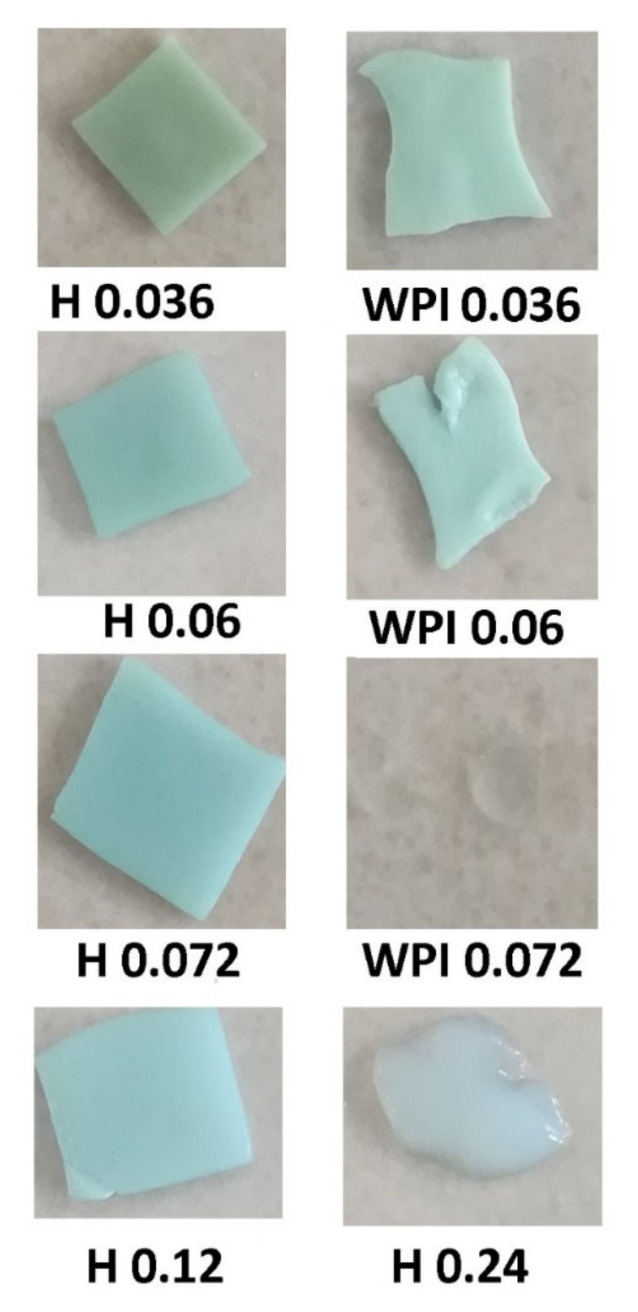
Photographs of hydrogel samples after a week spent in swelling environment.

The pictures of the samples after a week in aqueous solution (Figure 7) revealed that hydrogels containing WPI were very soft and subsequently very difficult to manipulate, looking more like gels, while the samples containing hydrolyzed whey proteins are much more stable. Samples with no copper could not be removed from tea bags with a gel-like structure. In the case of sample H 0.24 with a swelling degree of 10 g water/g, hydrogel the same gel consistency has been observed. One can conclude that crosslinking both trough ionic and hydrogen bonds stabilized the hydrogels, and the use of partially hydrolyzed whey proteins has a good influence on hydrogel stability.

Future research directions may also be involved in the studies related to the modifications in the secondary structures of proteins following hydrolysis and the synthesis of mixed hydrogels. Obtaining hydrogel composites was also taken into consideration.

## 4. Conclusions

We obtained a new type of hydrogel based on whey protein isolates (WPI), whey protein hydrolysates (WPH), and gelatin crosslinked with Cu^2+^ ions and plasticized with glycerol by a microwave-assisted method.

Enzymatic hydrolysis using bromelain led to changes in the structure of whey proteins, leading to an increase in the number of functional groups that have been involved in the crosslinking process.

The use of whey protein hydrolysates instead of whey protein isolates resulted in decreasing the swelling degree and increasing the hydrogel’s stability.

Increasing copper ion concentrations led to higher hydrogel stability, allowing higher swelling degrees. The best hydrogel samples lasted for one week in swelling medium (water).

The swelling process followed a second order kinetic.

## Figures and Tables

**Table 1:** The conditions for the obtaining of gelatin-whey hydrogels.

Sample Name	Grams CuSO_4_ *5H_2_O/Sample	Concentration ^1^ CuSO_4_ * 5H_2_O [%]	Sample Preparation	Average Films Thickness [μm]
H	0	0.0	0.5 g gelatin + 0.4 g glycerol + 4 mL hydrolysate + 2 mL water	231
H WPI	0	0.0	0.5 g gelatin + 0.4 g glycerol + 4 mL WPI 5% + 2 mL water	-
H 0.036	0.036	0.5	0.5 g gelatin + 0.4 g glycerol + 4 mL hydrolysate + 2 mL water + 0.036 g CuSO_4_ * 5H_2_O	266
WPI 0.036	0.036	0.5	0.5 g gelatin + 0.4 g glycerol + 4 mL WPI 5% + 2 mL water + 0.036 g CuSO_4_ * 5H_2_O	262
H 0.06	0.06	0.9	0.5 g gelatin + 0.4 g glycerol + 4 mL hydrolysate + 2 mL water + 0.06 g CuSO_4_ * 5H_2_O	286
WPI 0.06	0.06	0.9	0.5 g gelatin + 0.4 g glycerol + 4 mL WPI 5% + 2 mL water + 0.06 g CuSO_4 *_ 5H_2_O	290
H 0.072	0.072	1	0.5 g gelatin + 0.4 g glycerol + 4 mL hydrolysate + 2 mL water + 0.072 gCuSO_4_ * 5H_2_O	271
WPI 0.072	0.072	1	0.5 g gelatin + 0.4 g glycerol + 4 mL WPI 5% + 2 mL water + 0.072 gCuSO_4_ * 5H_2_O	269
H 0.12	0.12	1.7	0.5 g gelatin + 0.4 g glycerol + 4 mL hydrolysate 2 mL water + 0.12 g CuSO_4_ * 5H_2_O	269
H 0.24	0.24	3	0.5 g gelatin + 0.4 g glycerol + 4 mL hydrolysate 2 mL water + 0.24 g CuSO_4_ * 5H_2_O	278

^1^ Concentration CuSO_4_ * 5H_2_O [%] reported to sample without water.

**Table 2:** The variation of swelling degree (experimental and calculated) and rate constant.

Sample	H	H-WPI	H 0.036	WPI 0.036	H 0.06	WPI 0.06	H 0.072	WPI 0.072	H 0.12	H 0.24
n_F_	1.07	1.18	0.85	1.53	1.39	0.79	1.31	0.64	1.11	0.79
k_F_	1.97	1.77	1.66	2.66	2.48	1.33	2.28	1.40	2.06	1.50
R_F_^2^	0.7392	0.9997	0.9454	0.8894	0.9094	0.9704	0.8168	0.7137	0.9431	0.9326
SW calculated	3.66	2.90	3.05	4.08	2.70	6.98	5.18	5.60	8.33	9.99
SW experimental	3.61	2.55	3.03	4.70	2.69	6.97	4.70	5.56	8.26	9.19
k [1/h]	0.16	4.92	0.06	2.22	0.89	1.19	0.25	0.19	0.11	0.10
R^2^	0.9987	0.9999	0.9975	0.9996	0.9994	0.9999	0.9988	0.9995	0.9997	0.9998

## Data Availability

Not applicable.
